# Programmable Self-Assembly of Gold Nanoarrows via Regioselective Adsorption

**DOI:** 10.34133/2021/9762095

**Published:** 2021-07-28

**Authors:** Cheng Chen, Liheng Zheng, Fucheng Guo, Zheyu Fang, Limin Qi

**Affiliations:** ^1^Beijing National Laboratory for Molecular Sciences (BNLMS), College of Chemistry, Peking University, Beijing 100871, China; ^2^State Key Laboratory for Mesoscopic Physics, Collaborative Innovation Center of Quantum Matter, School of Physics, Peking University, Beijing 100871, China

## Abstract

Programing the self-assembly of colloidal nanoparticles into predetermined superstructures represents an attractive strategy to realize functional assemblies and novel nanodevices, but it remains a challenge. Herein, gold nanoarrows (GNAs) showing a distinct convex-concave structure were employed as unique building blocks for programmable self-assembly involving multiple assembly modes. Regioselective adsorption of 1,10-decanedithiol on the vertexes, edges, and facets of GNAs allowed for programmable self-assembly of GNAs with five distinct assembly modes, and regioselective blocking with 1-dodecanethiol followed by adsorption of 1,10-decanedithiol gave rise to programmable self-assembly with six assembly modes including three novel wing-engaged modes. The assembly mode was essentially determined by regioselective adsorption of the dithiol linker dictated by the local curvature together with the shape complementarity of GNAs. This approach reveals how the geometric morphology of nanoparticles affects their regioselective functionalization and drives their self-assembly.

## 1. Introduction

Solution-based self-assembly represents a powerful strategy for spontaneous organization of individual nanoparticles into well-defined assemblies (e.g., small clusters and ordered superstructures) that exhibit distinct collective properties [1–4]. Particularly, owing to strong surface plasmon coupling and light-matter coupling, plasmonic nanoparticle assemblies [5] have demonstrated immense potential in functional applications such as optoelectronic devices including surface enhanced Raman scattering (SERS) [4], biosensing and medical diagnostics [6, 7], chiral catalysis [7], and plasmonic metamaterials [8]. Since the properties of the nanoparticle ensembles are remarkably dependent on the shape, orientation, and spatial arrangement of the nanoparticles, precise control over the architectural configuration as well as the structural complexity is necessitated for achieving desired applications [9, 10]. In this regard, self-assembly of anisotropic nanoparticles is receiving increasing attention because the particle geometry plays a key role in engineering hierarchical assemblies with particular structures [11, 12]. For example, anisotropic nanocrystals with different morphologies, such as rods [4, 13, 14], plates [15], polyhedral [16–19], dumbbells [20], arrows [21], and multipods [22, 23], have been successfully employed for assembling superstructures with tunable and complicated architectures. Nevertheless, it remains challenging to program the self-assembly of colloidal nanoparticles into desirable structures with predetermined configurations [24, 25].

Programmable self-assembly of colloidal particles into sophisticated superstructures requires site-selective directional bonding or interactions [25, 26]. Generally, the shape anisotropicity in combination with regioselective functionalization can endow anisotropic nanoparticles with desirable bonding directionality needed for programmable self-assembly. As prototypical anisotropic nanoparticles, gold nanorods (GNRs) have been widely employed for site-selective functionalization to achieve end-to-end and side-by-side self-assembly selectively [11, 27, 28]. Notably, the local curvature is a crucial parameter in determining the site-selective adsorption of functional ligands on anisotropic nanocrystals. For example, thiolated ligands tend to preferentially bond to the two ends of cetyltrimethylammonium bromide- (CTAB-) capped GNRs at low concentrations because of the smaller packing density of CTAB at the ends with a larger curvature [29], which can lead to the end-to-end assembly of GNRs with the ends functionalized with either molecular ligands like cysteamine [30] or polymers like thiol-terminated polystyrene [31]. Interestingly, the preferential adsorption of 2-naphthalenethiols on the tips of gold triangular nanoprisms and subsequent attachment of polystyrene-*b*-poly-(acrylic acid) resulted in tip-patched nanoprisms that can be assembled into unusual twisted dimers [32]. Besides small molecules and polymers, DNA has been the most frequently used as powerful ligands to enable programmed self-assembly of nanoparticles into structures with increased complexity and diversity [33]. It is noteworthy that unprecedented complexity of DNA-programmed nanoparticle assemblies has been achieved based on two strategies: DNA-mediated self-assembly and DNA as a structure-defining element [34]. As an outstanding example of the DNA-programmed self-assembly, DNA-modified triangular gold bipyramids were successfully assembled into the most sophisticated clathrate architectures [17]. Considering that the nonselective binding of the terminal thiol of DNA to the different facets of anisotropic nanoparticles could result in a mixture of nonuniformly encoded nanoparticles, a regioselective surface encoding method based on selective blocking of particle surfaces with a polymer was recently developed [25]. Despite progress toward programmable self-assembly, the anisotropic nanoparticles employed as the building blocks are still largely limited to the particles with a convex geometry, and the complexity of the realized nanoparticle assemblies remains to be increased. Since information-rich building blocks are generally required for the self-assembly of complex structures [24], the unique nanoarrows with a distinct convex-concave structure and clearly demarcated surface curvatures [21, 35] would represent a promising building block for programmable self-assembly.

Herein, we demonstrate that regioselective adsorption of 1,10-decanedithiol dictated by the local curvature allows for programmable self-assembly of gold nanoarrows (GNAs) with five distinct assembly modes. Furthermore, a two-step self-assembly process involving regioselective blocking with 1-dodecanethiol followed by adsorption of 1,10-decanedithiol leads to six assembly modes including three novel wing-engaged modes. This approach reveals how the geometric morphology of nanoparticles affects their regioselective functionalization and drives their self-assembly, which represents a significant step toward programmable self-assembly of nanoparticles with designed shapes into diverse and complicated superstructures.

## 2. Results and Discussion

**Scheme 1:**
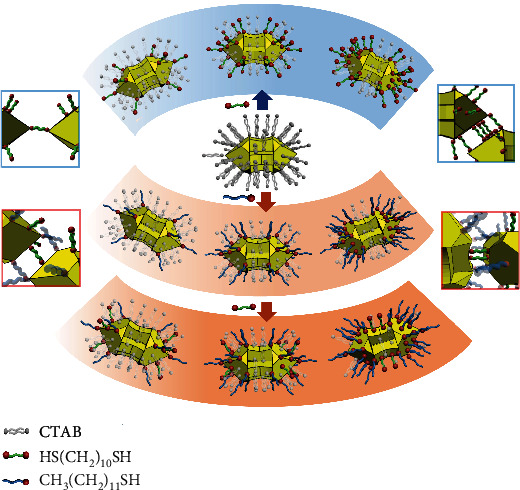
Schematic illustration of general self-assembly strategies based on regioselective adsorption on GNAs: (upper) one-step dithiol-induced self-assembly and (lower) two-step thiol-dithiol-induced self-assembly.

The unique [001]-elongated GNAs consist of two pyramidal heads exhibiting four {111} facets together with four truncated {100} facets, which are connected by a four-wing shaft with each wing parallel to {110} facets [21]. The well-defined convex-concave structure endows the GNAs with a variety of distinct surface curvatures; particularly, the local curvature decreases from positive for vertexes and edges through zero for facets to negative for the outer surface of wings [35]. The general self-assembly strategies based on regioselective adsorption on GNAs are schematically illustrated in Scheme 1. Initially, the GNAs are capped by CTAB bilayers that can be readily replaced by thiol-terminated molecules due to the strong Au-S bonding. Notably, thiolated ligands tend to replace the CTAB molecules on a location with a higher curvature due to a lower packing density of CTAB [29], indicating that there exists a preferential order for the regioselective adsorption: vertexes, edges, facets, and wings. In the one-step dithiol-induced self-assembly, 1,10-decanedithiol is introduced as a divalent linker, which would exclusively bond to the vertexes of GNAs at a low concentration, and sequentially bond to the edges and facets with increasing concentration, leading to a variety of assembly modes ranging from vertex-to-vertex to facet-to-facet. Alternatively, a two-step thiol-dithiol-induced self-assembly involves regioselective blocking of GNAs with the monovalent ligand 1-dodecanethiol and subsequent regioselective adsorption of the divalent linker 1,10-decanedithiol, leading to a variety of assembly modes ranging from edge-to-edge to wing-to-wing.

**Figure 1:**
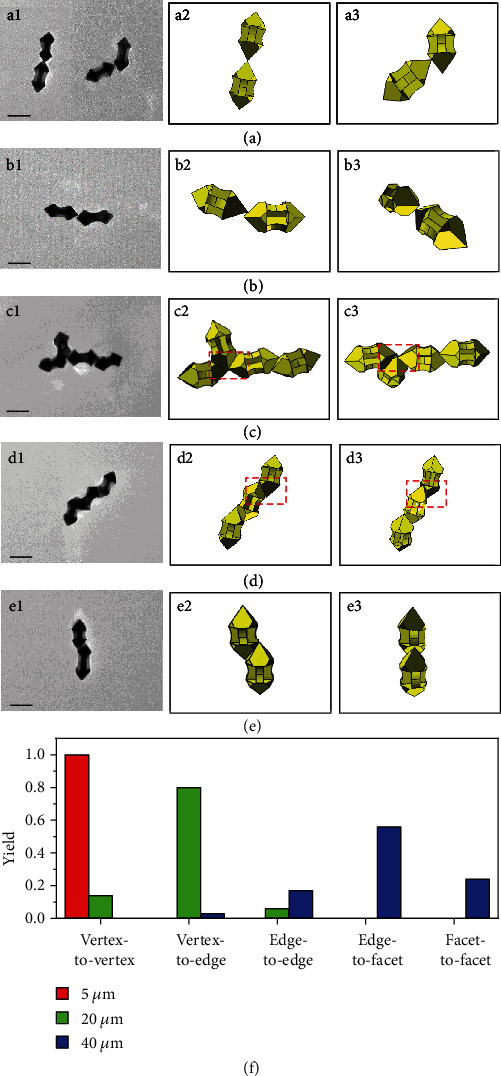
Assembly modes for one-step dithiol-induced self-assembly. TEM images (a1, b1, c1, d1, and e1) and geometric models (a2-3, b2-3, c2-3, d2-3, and e2-3) of vertex-to-vertex (a), vertex-to-edge (b), edge-to-edge (c), edge-to-facet (d), and facet-to-facet (e) assemblies. Framed boxes highlight the specific assembly mode. Scale bar: 100 nm. (f) Statistical analysis of yield of assembly modes at different dithiol concentrations.

Well-defined GNAs ~125 nm in length were used as nanoscale building blocks for self-assembly, and their spatial orientation can be easily determined based on the orientation of the central four-wing shaft as well as the apparent vertex angle of the pyramidal heads of the GNAs lying on the substrate (Figure S1). Through one-step dithiol-induced self-assembly, five distinct assembly modes can be achieved based on regioselective adsorption of the dithiol linker (Figure 1). Introduction of 5 *μ*M 1,10-decanedithiol to the GNAs dispersion induced self-assembly of the GNAs into regular GNA assemblies (termed as GNA-dithiol-5), as evidenced by the time evolution of the absorption spectra (Figure S2a), which revealed a gradual redshift of the longitudinal plasmon peak from 934 to 958 nm with increasing time to 2 h. Typical transmission electron microscopy (TEM) images suggested that the GNA-dithiol-5 exclusively adopted a vertex-to-vertex assembly mode (Figure 1(a1) and Figure S3), which basically consisted of two contact ways: top vertex-to-top vertex (Figure 1(a2)) and top vertex-to-side vertex (Figure 1(a3)). As the 1,10-decanedithiol concentration was increased to 20 *μ*M, in addition to the vertex-to-vertex mode, the obtained GNA-dithiol-20 exhibited two new assembly modes: vertex-to-edge (Figure 1(b)) and edge-to-edge (Figure 1(c)), and the vertex-to-edge became the dominant assembly mode (Figure S4). If the 1,10-decanedithiol concentration was further increased to 40 *μ*M, two novel assembly modes appeared for the resultant GNA-dithiol-40, i.e., edge-to-facet (Figure 1(d)) and facet-to-facet (Figure 1(e)), with the edge-to-facet as the most frequently observed assembly mode. It is noteworthy that the predominant number of the GNAs constituting the assemblies was generally increased from 2 for GNA-dithiol-5 (Figure S3) to 4-6 for GNA-dithiol-40 (Figure S5). This result is consistent with the time evolution of the absorption spectra of GNA-dithiol-40 (Figure S2b), which showed a larger redshift of the longitudinal plasmon peak from 934 to 984 nm, indicating a longer chain-like assemblies [29]. To quantify the yield of each assembly mode at different dithiol concentrations, a number of GNA assemblies involving more than 200 assembly sites connecting adjacent GNAs were statistically analyzed based on TEM observations (Figure 1(f)), which indicated a general change in the assembly mode from vertex-to-vertex through vertex-to-edge to edge-to-facet with increasing the dithiol concentration. Considering the possible perturbation of the preparation of TEM samples to the assembly configuration, the GNA assemblies were stabilized by silica coating in solution [36]. While the assembly modes at different dithiol concentrations were essentially unchanged, some complicated three-dimensional (3D) configurations could be captured for the silica-coated assemblies (Figure S6-9), indicating that the normal sample preparation did not considerably change the contact way of GNAs due to the strong bonding of the thiol group to gold despite possible deformation of the 3D configuration upon drying on copper grids. It is noteworthy that the SiO_2_ coating can well preserve the structure of nanoparticle assemblies formed in solution and endow them with long-term stability and solution processability [36, 37], which would allow for exploring useful applications of the stabilized GNA assemblies.

**Figure 2:**
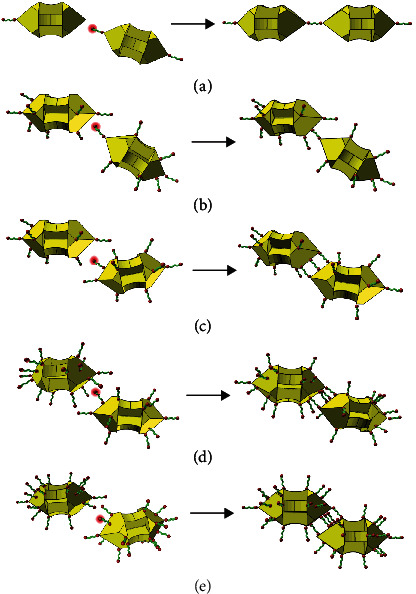
Schematic illustration of assembly mechanisms of one-step dithiol-induced self-assembly: (a) vertex-to-vertex, (b) vertex-to-edge, (c) edge-to-edge, (d) edge-to-facet, and (e) facet-to-facet. For simplicity, the adsorbed CTAB molecules are omitted. The thiol groups to bond to another GNA are highlighted with red halos.

The observed assembly modes of the one-step dithiol-induced self-assembly of GNAs can be rationalized in terms of regioselective adsorption of 1,10-decanedithiol dictated by the local curvature (Figure 2). Since the vertexes of GNAs have the highest local curvatures, the dithiol linker would be preferentially adsorbed on the top and side vertexes at a low concentration (e.g., 5 *μ*M). Therefore, the thiol end group of the dithiol ligand attached to a vertex of a GNA would tend to bond to an available vertex of another GNA, where the dithiol ligand was not adsorbed or was unsaturatedly adsorbed, resulting in the exclusive vertex-to-vertex assembly mode (Figure 2(a) and Figure S10). The observation that the GNA dimmers were the predominant product indicated that the amount of the dithiol linker could be too low to occupy all the vertexes, and hence, the further aggregation would be prevented owing to the lack of dithiol molecules. When the dithiol concentration was increased to 20 *μ*M, most of the vertexes were occupied by dithiol and a few sites on the edges were also attached by dithiol, leading to occurrence of the vertex-to-edge mode (Figure 2(b)) and the edge-to-edge mode (Figure 2(c)). For the edge-to-edge mode, the bonding of a thiol end group of the dithiol adsorbed on an edge to a blank edge site of another GNA would be accompanied by subsequent bonding of the neighbouring dithiol linkers to the blank sites on the edge, resulting in a stable edge-to-edge connection. With further increasing the dithiol concentration to 40 *μ*M, most of the vertexes and edges were occupied by dithiol and a few sites on the facets were also adsorbed by dithiol, leading to the appearance of the edge-to-facet mode (Figure 2(d)) and the facet-to-facet mode (Figure 2(e)). It may be noted that the adsorption of the dithiol linker and the self-assembly of the linker-attached GNAs occurred almost simultaneously; the coexistence of several assembly modes seemed inevitable for the self-assembly at a high dithiol concentration.

**Figure 3:**
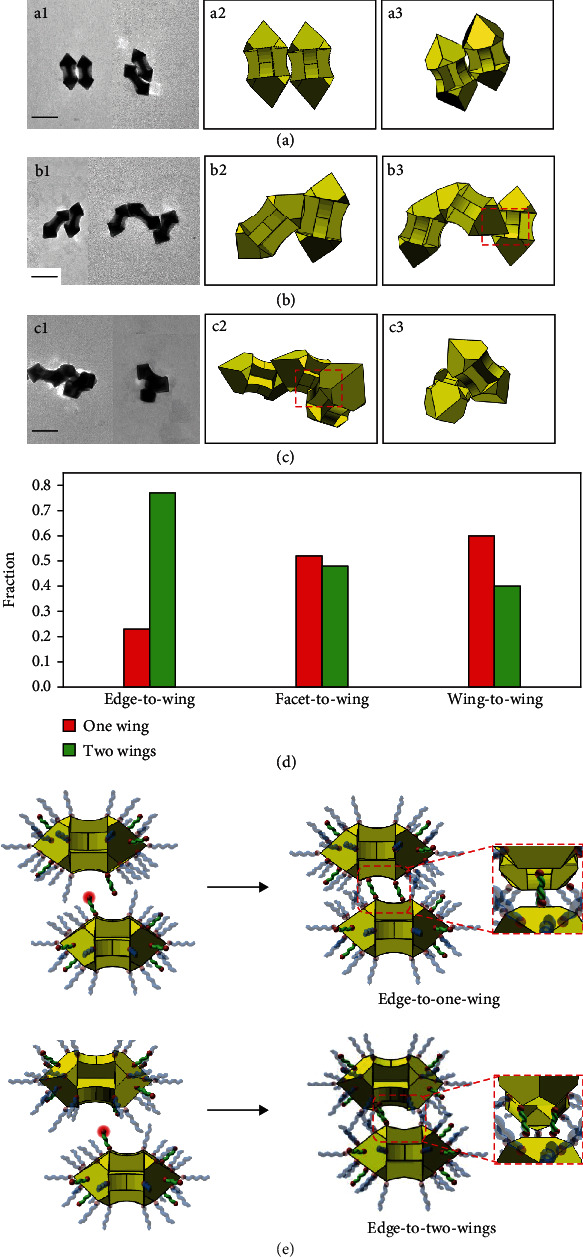
Wing-engaged assembly modes for two-step thiol-dithiol-induced self-assembly. TEM images (a1, b1, and c1) and geometric models (a2-3, b2-3, and c2-3) of edge-to-wing (a), facet-to-wing (b), and wing-to-wing (c) assemblies. Framed boxes highlight the specific assembly mode. Scale bar: 100 nm. (d) Statistical analysis of fraction of two contact ways in wing-engaged assembly modes. (e) Schematic illustration of assembly mechanisms of edge-to-one-wing and edge-to-two-wings modes. For simplicity, the adsorbed CTAB molecules are omitted. The thiol groups to bond to another GNA are highlighted with red halos.

If monocovalent 1-dodecanethiol was firstly introduced to the GNA dispersion before the divalent 1,10-decanedithiol was added, regioselective blocking of the GNAs occurred and subsequent adsorption of the dithiol linker could take place only on the unblocking areas. This two-step thiol-dithiol-induced self-assembly of GNAs could bring about novel assembly models. The concentration of 1-dodecanethiol was varied to change the blocking area while the concentration of the dithiol linker was kept at 10 *μ*M. When 20 *μ*M 1-dodecanethiol was introduced, the obtained GNA assemblies (termed as GNA-thiol-20-dithiol) exhibited three assembly modes, namely, edge-to-edge, edge-to-facet, and facet-to-facet (Figures S11 and S12). All these three modes had been observed in the GNA assemblies obtained by the one-step dithiol-induced assembly. Notably, the vertex-engaged modes (i.e., vertex-to-vertex and vertex-to-edge) did not appear at all because the vertexes had been completely blocked by the monothiol ligand, as schematically illustrated (Figure S13). With increasing the 1-dodecanethiol concentration to 40 *μ*M, considerable edge areas were blocked and the edge-to-wing emerged as the main assembly mode for the resultant GNA-thiol-40-dithiol (Figure 3(a) and Figure S14). Interestingly, this edge-to-wing mode basically contained two contact ways, namely, one pyramid edge contact one wing on its face (Figure 3(a2)) and one edge contact two wings on their edges (Figure 3(a3)). When the 1-dodecanethiol concentration was further increased to 60 *μ*M, the edges were largely blocked and the facet-to-wing mode (Figure 3(b)) emerged as the main assembly mode for the resultant GNA-thiol-60-dithiol, which was accompanied by the appearance of the wing-to-wing mode (Figure 3(c)).

**Figure 4:**
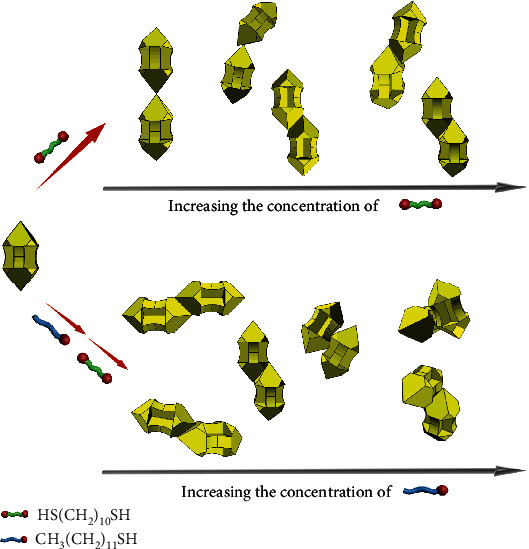
Schematic illustration of representative assembly modes of GNAs achieved by one-step dithiol-induced self-assembly and two-step thiol-dithiol-induced self-assembly.

It may be noted that for each of the three wing-engaged assembly modes, there existed two contact ways: one-wing-engaged and two-wing-engaged (Figures 3(a)–3(c) and Figure S15). The statistical analysis of the overall fractions of the two contact ways suggested that while the one-wing-engaged way was slightly preferred for the facet-to-wing and wing-to-wing modes, the two-wing-engaged way was significantly favored for the edge-to-wing mode (Figure 3(d)). Based on the proposed assembly mechanisms (Figure 3(e) and Figure S16), a pair of parallel GNAs adopting the edge-to-wing mode showed a high degree of shape complementarity and the two GNAs had a denser packing compared with the other two wing-engaged modes. It is known that for two nanoparticles approaching each other at a small separation distance, the closer they are, the higher the van der Waals (vdW) attraction energy becomes [1, 35]. Since the neighbouring GNAs with the edge-to-two-wings manner had a closer contact than the edge-to-one-wing manner, the strong vdW attraction would considerably contribute to the edge-to-two-wings assembly manner. Generally, with increasing the monothiol concentration in the two-step thiol-dithiol-induced self-assembly, the main assembly mode evolved from edge-to-facet through edge-to-wing to facet-to-wing (Figure S17). In summary, based on regioselective adsorption of thiolated ligands, five distinct assembly modes were achieved by one-step dithiol-induced self-assembly of GNAs, and six assembly modes including three novel wing-engaged modes were realized via two-step thiol-dithiol-induced self-assembly (Figure 4).

**Figure 5:**
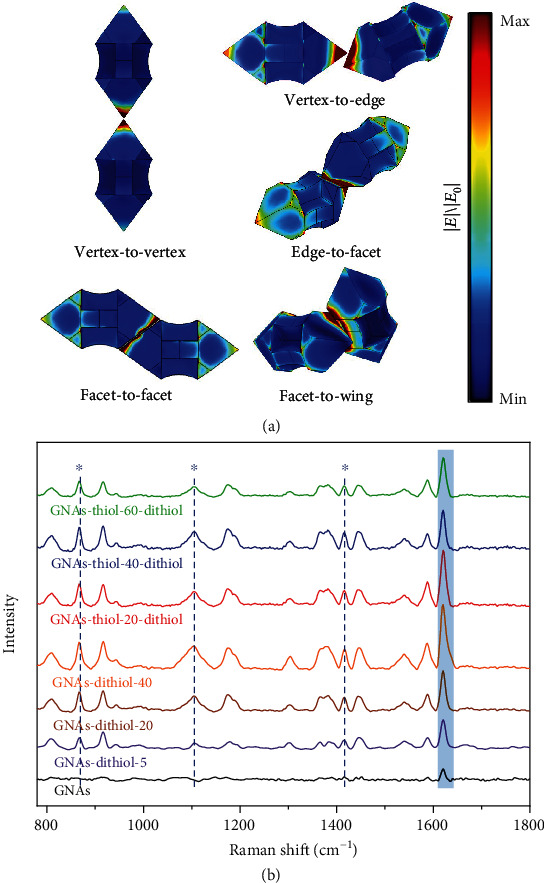
(a) Simulation of electric field distribution for five typical assembly modes. (b) Raman spectra of CV adsorbed on GNAs and GNA assemblies dispersed in DMF. “∗” denotes the peaks from the solvent DMF.

The obtained diverse GNA assemblies represent unique plasmonic nanostructures showing notable structure-dependent field confinement. The electric field distribution of GNA dimers with five representative assembly modes was simulated with a finite element method (FEM), which displayed a variety of hot spots generated from strong localized surface plasmon resolution (LSPR) coupling among adjacent GNAs (Figure 5(a)). It is noteworthy that the contact location of the assembly adopting the vertex-to-edge mode exhibited the highest normalized electric field intensity among the five assemblies probably owing to the existence of strong tip-edge-tip plasmon coupling. It is known that the hotspots located at the nanogaps between plasmonic nanoparticles as well as the sharp corners of the particles contribute greatly to SERS [38]. The distinct nanogaps between adjacent nanoarrows with various contact manners together with plenty of sharp tips and edges of each nanoarrow would endow the GNAs with strong SERS response toward probe molecules. Crystal violet (CV) was employed as a probe molecule to evaluate the SERS property of the different GNA assemblies dispersed in DMF. The CV molecules exhibited a strong peak at 1620 cm^−1^ attributed to the aromatic C–C stretching vibration [39], and its intensity displayed significant difference for the GNA assemblies obtained under different conditions (Figure 5(b)). Particularly, the intensities for GNA-dithiol-5, GNA-dithiol-20, and GNA-dithiol-40 were 2.8-fold, 3.8-fold, and 6.0-fold higher than those for dispersed GNAs, respectively. This result indicated that the SERS performance of the GNA assemblies was generally increased from vertex-to-vertex through vertex-to-edge to edge-to-facet and facet-to-facet, which was associated with increased contact area and augmented hotspot amount. Among the GNA assemblies obtained by the two-step thiol-dithiol-induced self-assembly, the GNA-thiol-20-dithiol with mainly the edge-to-facet and facet-to-facet modes showed the strongest Raman intensity, confirming the importance of the contact area or the hotspot amount [40] in determining the SERS performance of GNA assemblies. These results suggested that tunable plasmonic characteristics and SERS properties could be readily achieved by manipulating the self-assembly mode via simply changing the ligand concentration, indicating potential applications in optoelectronics, biosensing, and biomedicine.

## 3. Conclusion

In conclusion, regioselective adsorption of 1,10-decanedithiol on the vertexes, edges, and facets of GNAs allowed for programmable self-assembly of GNAs with five distinct assembly modes, and regioselective blocking with 1-dodecanethiol followed by adsorption of 1,10-decanedithiol gave rise to programmable self-assembly with six assembly modes including three novel wing-engaged modes. It was unraveled that the assembly mode was essentially determined by regioselective adsorption of dithiol linkers dictated by the local curvature together with the shape complementarity of GNAs. The fact that multiple complex assemblies were readily obtained by introducing simple small molecular ligand demonstrated the superiority of nanoarrows with multiple distinct surface curvatures as unique building blocks. This approach revealed how the geometric morphology and regioselective functionalization of nanoparticles drove their self-assembly, which represents a significant step toward programmable self-assembly of nanoparticles with designed shapes into diverse and complicated superstructures.

## 4. Materials and Methods

### 4.1. Materials

Cetyltrimethylammonium bromide (CTAB, ≥98%) and cetyltrimethylammonium chloride (CTAC, 25 wt % in H_2_O) were obtained from Sigma-Aldrich, Co. Silver nitrate (AgNO_3_, >99%), sodium borohydride (NaBH_4_, 99%), L-ascorbic acid (AA, >99%), and crystal violet (CV) were obtained from Sinopharm Chemical Reagent Co. Ltd. Hydrochloroauric acid trihydrate (HAuCl_4_·3H_2_O, 99.9%) was from Beijing Chemical Reagents Co. N,N-Dimethylformamide (DMF) was purchased from Tianjin Concord Technology Co. 1,10-Decanedithiol was obtained from Alfa Aesar Co., and 1-dodecanethiol was obtained from Aladdin Co. Tetraethyl orthosilicate (TEOS, 98%) was from Shantou Xilong Chemical Factory. Ammonia water was from Beijing Tongguang Fine Chemicals Co. The water was deionized.

### 4.2. Instrumentation

UV-vis spectra were measured using a UV-vis spectrophotometer (Hitachi U-4100). Transmission electron microscopy (TEM) observations were performed on FEI Tecnai T20 at 200 kV, and scanning electron microscope (SEM) observations were performed using Hitachi S-4800 at 5–10 kV. Raman spectra were recorded on a Raman spectrometer (Thermo Fisher Scientific-DXRxi) using an incident laser of 532 nm.

### 4.3. One-Step Dithiol-Induced Self-Assembly of GNAs

The GNAs were prepared through overgrowth of the produced GNRs [41] following the previously reported procedure [21] (see the details in Supplementary Materials). The GNAs dispersed in 0.5 mM CTAB were centrifuged at 6000 rpm for 4 min and then redispersed in DMF, resulting in a concentrated GNA dispersion with a particle concentration (~6 nM). A certain amount of 1 mM 1,10-decanedithiol solution in DMF was injected into 1 mL of the GNA dispersion in DMF to give a certain concentration (typically, 5 *μ*M, 20 *μ*M, and 40 *μ*M) under vigorous stirring for 8 min, and then, the solution was kept undisturbed for 2 h, which was accompanied by a gradual color change from reddish to purple-gray, indicating the formation of GNA assemblies in solution. A drop of the resultant dispersion was dropped on a copper grid placed on filter paper and quickly dried in air for further TEM examination.

### 4.4. Two-Step Thiol-Dithiol-Induced Self-Assembly of GNAs

Firstly, a certain amount of 1 mM 1-dodecanethiol solution in DMF was added into 1 mL of the concentrated GNA dispersion in DMF with stirring for 8 min, giving a certain 1-dodecanethiol concentration (typically, 20 *μ*M, 40 *μ*M, and 60 *μ*M). The solution was kept undisturbed for 3 h, which was followed by centrifugation at 6000 rpm for 4 min to remove the extra 1-dodecanethiol and redispersion in DMF. Secondly, 1,10-decanedithiol (10 *μ*L, 1 mM) was injected into the dispersion under stirring for 8 min and incubated for 2 h, resulting in the self-assembly of the GNAs functionalized by 1-dodecanethiol. A drop of the resultant dispersion was dropped on a copper grid placed on a filter paper and quickly dried in air for further TEM examination.

### 4.5. Silica Coating of GNA Assemblies

The silica coating of the GNA assemblies were achieved by ammonia-catalyzed hydrolysis of TEOS following the reported method [36] with minor modification. TEOS solution in ethanol (10 *μ*L, 10 vol%) was injected into the assembly solution after ammonia water (140 *μ*L, 25 wt%) was added under vigorous stirring. The reaction mixture was stirred for another 30 min, and then, the product was collected by centrifugation and redispersed in ethanol. A drop of the dispersion was dried in air at room temperature on a copper grid for TEM examination.

### 4.6. SERS Measurements

Crystal violet (200 *μ*L, 1 mM), the Raman probe molecule, was added into the assembly solution and left undisturbed for at least 12 h for adequate adsorption. Then, the solution was centrifuged at 5000 rpm for 4 min, and the solid was redispersed in DMF (~1 mL). A drop of the dispersion was placed on a glass slide for the SERS measurements, which were performed by fine focusing a 10x microscope objective with a laser spot diameter of 1 *μ*m.

### 4.7. Electromagnetic Simulation

COSMOL multiphysics software was used to simulate the optical response of the GNA assemblies based on FEM, with the size of the nanogap between adjacent GNAs set as 3 nm. The permittivity of gold was acquired from the reported data [42]. The excitation wavelength for the vertex-to-vertex, vertex-to-edge, edge-to-facet, facet-to-facet, and facet-to-wing assemblies was 860 nm, 740 nm, 855 nm, 835 nm, and 780 nm, respectively, which were the positions of maximum absorption obtained by simulation, respectively.

## Data Availability

All data needed to evaluate the conclusions in the paper are present in the paper and/or the Supplementary Materials. Additional data related to this paper may be requested from the authors.

## Supplementary Materials

Supplementary Materials Experimental details for synthesis of GNAs. Figure S1: SEM and TEM images of GNAs. Figure S2: time evolution of absorption spectra of GNA-dithiol-5 and GNA-dithiol-40 after the introduction of 1,10-decanedithiol. Figure S3: TEM image of GNA-dithiol-5. Figure S4: TEM images of GNA-dithiol-20. Figure S5: TEM images of GNA-dithiol-40. Figure S6: TEM images and geometric models of one-step dithiol-induced GNA assemblies after silica coating. Figure S7: TEM images of silica-coated GNA-dithiol-5. Figure S8: TEM images of silica-coated GNA-dithiol-20. Figure S9: TEM images of silica-coated GNA-dithiol-40. Figure S10: schematic illustration of one-step dithiol-induced self-assembly of GNAs in a top vertex-to-side vertex mode. Figure S11: TEM images and geometric models of edge-to-edge, edge-to-facet, and facet-to-facet assemblies obtained by two-step thiol-dithiol-induced self-assembly of GNAs. Figure S12: TEM images of GNA-thiol-20-dithiol. Figure S13: schematic illustration of assembly mechanisms of two-step thiol-dithiol-induced self-assembly: edge-to-edge, edge-to-facet, and facet-to-facet. Figure S14: TEM images of GNA-thiol-40-dithiol. Figure S15: TEM images of GNA-thiol-60-dithiol. Figure S16: schematic illustration of assembly mechanisms of two-step thiol-dithiol-induced self-assembly: facet-to-one-wing, facet-to-two-wings, wing-to-one-wing, and wing-to-two-wings. Figure S17: statistical analysis of the yield of assembly modes for two-step thiol-dithiol-induced self-assembly at different thiol concentrations.

## References

[1] Boles M. A., Engel M., Talapin D. V. (2016). Self-assembly of colloidal nanocrystals: from intricate structures to functional materials. *Chemical Reviews*.

[2] Grzelczak M., Liz-Marzán L. M., Klajn R. (2019). Stimuli-responsive self-assembly of nanoparticles. *Chemical Society Reviews*.

[3] Chen L., Su B., Jiang L. (2019). Recent advances in one-dimensional assembly of nanoparticles. *Chemical Society Reviews*.

[4] Wei W., Bai F., Fan H. (2019). Oriented gold nanorod arrays: self-assembly and optoelectronic applications. *Angewandte Chemie, International Edition*.

[5] Klinkova A., Choueiri R. M., Kumacheva E. (2014). Self-assembled plasmonic nanostructures. *Chemical Society Reviews*.

[6] Zhao Y., Xu C. (2020). DNA-based plasmonic heterogeneous nanostructures: building, optical responses, and bioapplications. *Advanced Materials*.

[7] Mosquera J., Zhao Y., Jang H.-J. (2020). Plasmonic nanoparticles with supramolecular recognition. *Advanced Functional Materials*.

[8] Mueller N. S., Okamura Y., Vieira B. G. M. (2020). Deep strong light-matter coupling in plasmonic nanoparticle crystals. *Nature*.

[9] Yi C., Liu H., Zhang S. (2020). Self-limiting directional nanoparticle bonding governed by reaction stoichiometry. *Science*.

[10] Zhou W., Liu Z., Huang Z. (2020). Device-quality, reconfigurable metamaterials from shape-directed nanocrystal assembly. *Proceedings National Academy of Sciences United States of America*.

[11] Zhang S.-Y., Regulacio M. D., Han M.-Y. (2014). Self-assembly of colloidal one-dimensional nanocrystals. *Chemical Society Reviews*.

[12] Deng K., Luo Z., Tan L., Quan Z. (2020). Self-assembly of anisotropic nanoparticles into functional superstructures. *Chemical Society Reviews*.

[13] Lan X., Su Z., Zhou Y. (2017). Programmable supra-assembly of a DNA surface adapter for tunable chiral directional self-assembly of gold nanorods. *Angewandte Chemie, International Edition*.

[14] Lu J., Xue Y., Bernardino K. (2021). Enhanced optical asymmetry in supramolecular chiroplasmonic assemblies with long-range order. *Science*.

[15] Ye X., Chen J., Engel M. (2013). Competition of shape and interaction patchiness for self-assembling nanoplates. *Nature Chemistry*.

[16] Henzie J., Grünwald M., Widmer-Cooper A., Geissler P. L., Yang P. (2012). Self-assembly of uniform polyhedral silver nanocrystals into densest packings and exotic superlattices. *Nature Materials*.

[17] Lin H., Lee S., Sun L. (2017). Clathrate colloidal crystals. *Science*.

[18] Nagaoka Y., Tan R., Li R. (2018). Superstructures generated from truncated tetrahedral quantum dots. *Nature*.

[19] Lee Y. H., Shi W., Yang Y. (2020). Modulating orientational order to organize polyhedral nanoparticles into plastic crystals and uniform metacrystals. *Angewandte Chemie, International Edition*.

[20] Liu Y., Deng K., Yang J. (2020). Shape-directed self-assembly of nanodumbbells into superstructure polymorphs. *Chemical Science*.

[21] Wang Q., Wang Z., Li Z. (2017). Controlled growth and shape-directed self-assembly of gold nanoarrows. *Science Advances*.

[22] Huang T., Zhao Q., Xiao J., Qi L. (2010). Controllable self-assembly of PbS nanostars into ordered structures: close-packed arrays and patterned arrays. *ACS Nano*.

[23] Miszta K., de Graaf J., Bertoni G. (2011). Hierarchical self-assembly of suspended branched colloidal nanocrystals into superlattice structures. *Nature Materials*.

[24] Cademartiri L., Bishop K. J. M. (2015). Programmable self-assembly. *Nature Materials*.

[25] Chen G., Gibson K. J., Liu D. (2019). Regioselective surface encoding of nanoparticles for programmable self-assembly. *Nature Materials*.

[26] Liu M., Zheng X., Grebe V., Pine D. J., Weck M. (2020). Tunable assembly of hybrid colloids induced by regioselective depletion. *Nature Materials*.

[27] Wang L., Zhu Y., Xu L. (2010). Side-by-side and end-to-end gold nanorod assemblies for environmental toxin sensing. *Angewandte Chemie, International Edition*.

[28] Xiao J., Qi L. (2020). Controllable self-assembly of gold nanorods via host–guest interaction between cyclodextrins and surfactants. *Acta Physico-Chimica Sinica*.

[29] Chen H., Shao L., Li Q., Wang J. (2013). Gold nanorods and their plasmonic properties. *Chemical Society Reviews*.

[30] Tan S. F., Anand U., Mirsaidov U. (2017). Interactions and attachment pathways between functionalized gold nanorods. *ACS Nano*.

[31] Nie Z., Fava D., Kumacheva E., Zou S., Walker G. C., Rubinstein M. (2007). Self-assembly of metal-polymer analogues of amphiphilic triblock copolymers. *Nature Materials*.

[32] Kim A., Zhou S., Yao L. (2019). Tip-patched nanoprisms from formation of ligand islands. *Journal of the American Chemical Society*.

[33] Si K. J., Chen Y., Shi Q., Cheng W. (2018). Nanoparticle superlattices: the roles of soft ligands. *Advancement of Science*.

[34] Jiang S., Zhang F., Yan H. (2020). Complex assemblies and crystals guided by DNA. *Nature Materials*.

[35] Liu C., Ou Z., Guo F. (2020). “Colloid–atom duality” in the assembly dynamics of concave gold nanoarrows. *Journal of the American Chemical Society*.

[36] Yin Z., Zhang W., Fu Q. (2014). Construction of stable chainlike Au nanostructures via silica coating and exploration for potential photothermal therapy. *Small*.

[37] Auyeung E., Macfarlane R. J., Choi C. H. J., Cutler J. I., Mirkin C. A. (2012). Transitioning DNA-engineered nanoparticle superlattices from solution to the solid state. *Advanced Materials*.

[38] Ding S.-Y., Yi J., Li J.-F. (2016). Nanostructure-based plasmon-enhanced Raman spectroscopy for surface analysis of materials. *Nature Reviews Materials*.

[39] Tang J., Ou Q., Zhou H., Qi L., Man S. (2019). Seed-mediated electroless deposition of gold nanoparticles for highly uniform and efficient sers enhancement. *Nanomaterials*.

[40] Wang Q., Li D., Xiao J., Guo F., Qi L. (2019). Reversible self-assembly of gold nanorods mediated by photoswitchable molecular adsorption. *Nano Research*.

[41] Ye X., Zheng C., Chen J., Gao Y., Murray C. B. (2013). Using binary surfactant mixtures to simultaneously improve the dimensional tunability and monodispersity in the seeded growth of gold nanorods. *Nano Letters*.

[42] Johnson P. B., Christy R. W. (1972). Optical constants of the noble metals. *Physical Review B*.

