# Supplementary material for: Programmable Self-Assembly of Gold Nanoarrows via Regioselective Adsorption
Source: Research (Wash D C). 2021 Jul 28;2021:9762095. doi: 10.34133/2021/9762095 (PMC8343431; doi:10.34133/2021/9762095)
Supplement: Supplementary Materials — Experimental details for synthesis of GNAs. Figure S1: SEM and TEM images of GNAs. Figure S2: time evolution of absorption spectra of GNA-dithiol-5 and GNA-dithiol-40 after the introduction of 1,10-decanedithiol. Figure S3: TEM image of GNA-dithiol-5. Figure S4: TEM images of GNA-dithiol-20. Figure S5: TEM images of GNA-dithiol-40. Figure S6: TEM images and geometric models of one-step dithiol-induced GNA assemblies after silica coating. Figure S7: TEM images of silica-coated GNA-dithiol-5. Figure S8: TEM images of silica-coated GNA-dithiol-20. Figure S9: TEM images of silica-coated GNA-dithiol-40. Figure S10: schematic illustration of one-step dithiol-induced self-assembly of GNAs in a top vertex-to-side vertex mode. Figure S11: TEM images and geometric models of edge-to-edge, edge-to-facet, and facet-to-facet assemblies obtained by two-step thiol-dithiol-induced self-assembly of GNAs. Figure S12: TEM images of GNA-thiol-20-dithiol. Figure S13: schematic illustration of assembly mechanisms of two-step thiol-dithiol-induced self-assembly: edge-to-edge, edge-to-facet, and facet-to-facet. Figure S14: TEM images of GNA-thiol-40-dithiol. Figure S15: TEM images of GNA-thiol-60-dithiol. Figure S16: schematic illustration of assembly mechanisms of two-step thiol-dithiol-induced self-assembly: facet-to-one-wing, facet-to-two-wings, wing-to-one-wing, and wing-to-two-wings. Figure S17: statistical analysis of the yield of assembly modes for two-step thiol-dithiol-induced self-assembly at different thiol concentrations. [file 9762095.f1.pdf]

Supplementary Materials for

Programmable Self-Assembly of Gold Nanoarrows via Regioselective Adsorption

Cheng Chen[1], Liheng Zheng[2], Fucheng Guo1, Zheyu Fang[2], and Limin Qi[1*]

[1]*Beijing National Laboratory for Molecular Sciences (BNLMS), College of Chemistry, Peking University, Beijing 100871, China*

[2]*State Key Laboratory for Mesoscopic Physics, Collaborative Innovation Center of Quantum Matter, School of Physics, Peking University, Beijing 100871, China*

Contents:

Experimental details for synthesis of GNAs

Figure S1. SEM and TEM images of GNAs.

Figure S2. Time evolution of absorption spectra of GNA-dithiol-5 and GNA-dithiol-40 after the introduction of 1,10-decanedithiol.

Figure S3. TEM image of GNAs-dithiol-5.

Figure S4. TEM images of GNAs-dithiol-20.

Figure S5. TEM images of GNAs-dithiol-40.

Figure S6. TEM images and geometric models of one-step dithiol-induced GNA assemblies after silica coating.

Figure S7. TEM images of silica-coated GNAs-dithiol-5.

Figure S8. TEM images of silica-coated GNAs-dithiol-20.

Figure S9. TEM images of silica-coated GNAs-dithiol-40.

Figure S10. Schematic illustration of one-step dithiol-induced self-assembly of GNAs in a top vertex-to-side vertex mode.

Figure S11. TEM images and geometric models of edge-to-edge, edge-to-facet, and facet-to-facet assemblies obtained by two-step thiol-dithiol-induced self-assembly of GNAs.

Figure S12. TEM images of GNAs-thiol-20-dithiol.

Figure S13. Schematic illustration of assembly mechanisms of two-step thiol-dithiol-induced self-assembly: edge-to-edge, edge-to-facet, and facet-to-facet.

Figure S14. TEM images of GNAs-thiol-40-dithiol.

Figure S15. TEM images of GNAs-thiol-60-dithiol.

Figure S16. Schematic illustration of assembly mechanisms of two-step thiol-dithiol-induced self-assembly: facet-to-one-wing, facet-to-two-wings, wing-to-one-wing, and wing-to-two-wings.

Figure S17. Statistical analysis of yield of assembly modes for two-step thiol-dithiol-induced self-assembly at different thiol concentrations.

Experimental details for synthesis of GNAs

Gold nanorods (GNRs) was first synthesized out using a seed-mediated method according to previous work [42] with minor modification. The seed solution was prepared by adding freshly prepared ice-cold $NaBH_4$ (600 μL, 10 mM) into a mixture of $HAuCl_4$ solution (250 μL, 10 mM) and CTAB solution (10 mL, 100 mM) under vigorous stirring. The solution color changed from yellow to brown after 20 s of stirring, and was aged at 30°C for 30 min. For the preparation of the growth solution, an aqueous sodium oleate (NaOL) solution (50 mL, 20 mM) was mixed with CTAB solution (38 mL, 100 mM) and water (13 mL), and then $AgNO_3$ solution (1.92 mL, 10 mM) was injected into the mixture under mild stirring, which was then kept undisturbed at 30°C for 15 min. Afterward, an aqueous $HAuCl_4$ solution (5 mL, 10 mM) was added into the mixed solution, which was then incubated at 30°C for 100 min. An aqueous ascorbic acid (AA) solution (5 mL, 10 mM) was added into the solution, and the mixture was kept vigorously stirring for 30 s, which was followed by injecting 80 μL of the seed solution into the growth solution. The resulting mixture was left undisturbed at 30°C overnight, separated by centrifugation, and redispersed into 1 mM CTAB solution for further use. Then, GNAs were synthesized via overgrowth of the prepared GNRs following a previously reported procedure [21]. An aqueous $HAuCl_4$ solution (3 mL, 10 mM) was firstly injected into a CTAC solution (100 mL, 100 mM) followed by sequential adding of $AgNO_3$ solution (1.5 mL, 10 mM) , HCl (2 mL, 1 M) and AA solution (1.5 mL, 100 mM) under mild stirring, leading to the formation of a growth solution. Then, 5 mL of 20-fold concentrated GNR seed solution was added into the growth solution. The reaction mixture was vigorously stirred for 10 s and left undisturbed at 30°C for ~ 4 hours. Then, it was purified by centrifugation and redispersed in 1 mM CTAB solution for the following assembly processes.

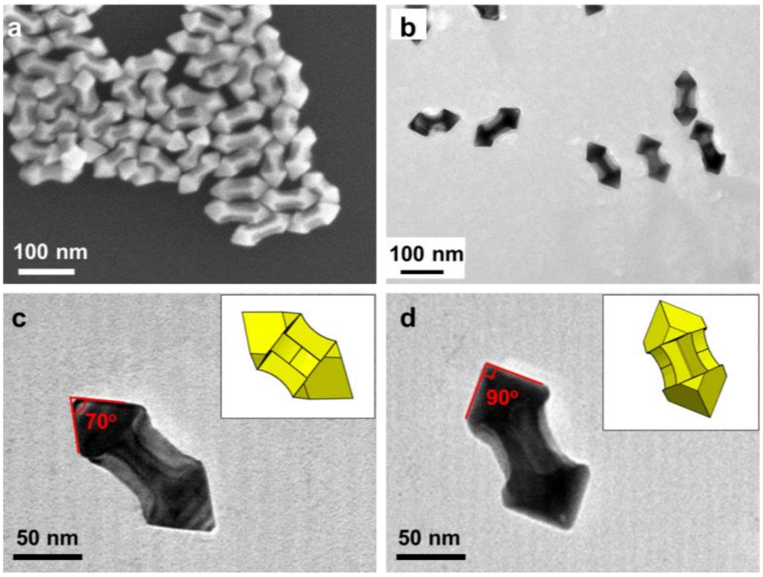

Figure S1. SEM (a) and TEM (b-d) images of GNAs. GNAs lying on the bottom edges (c) and the bottom side vertexes (d) of the square head pyramids exhibit their characteristic vertex angles.

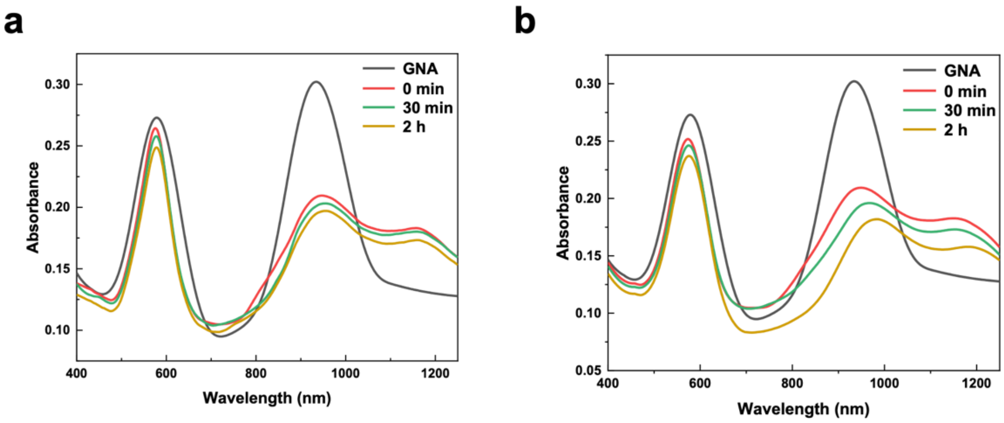

Figure S2. Time evolution of absorption spectra of (a) GNA-dithiol-5 and (b) GNA-dithiol-40 after the introduction of 1,10-decanedithiol. The absorption spectra of the original GNA dispersion are also shown.

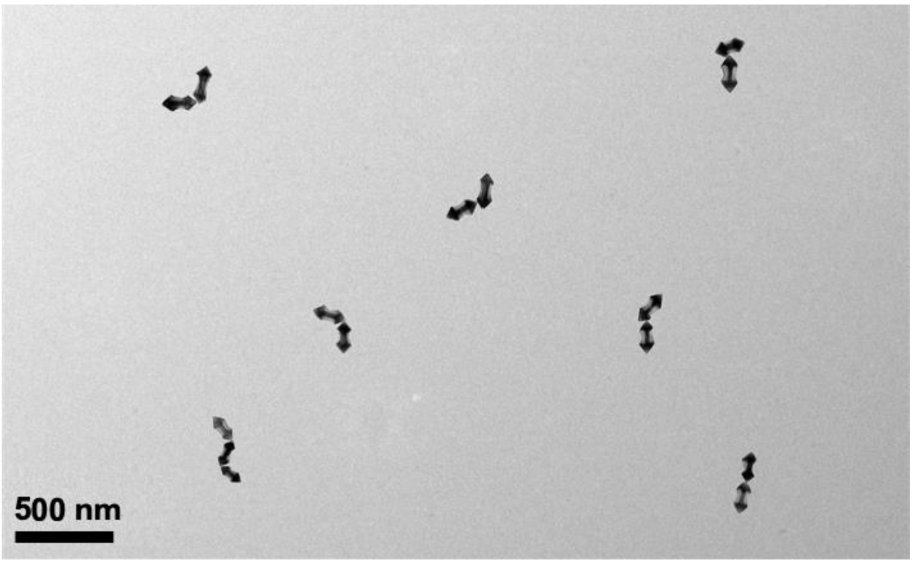

Figure S3. TEM image of GNAs-dithiol-5.

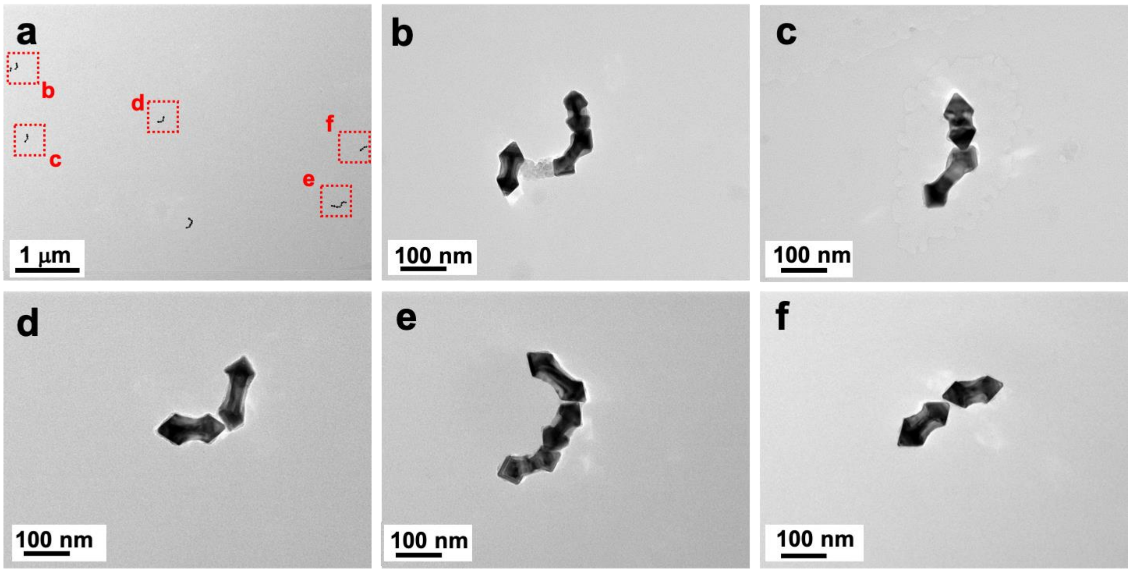

Figure S4. TEM images of GNAs-dithiol-20. Panels b-f present the high-magnification images of the framed areas in panel a.

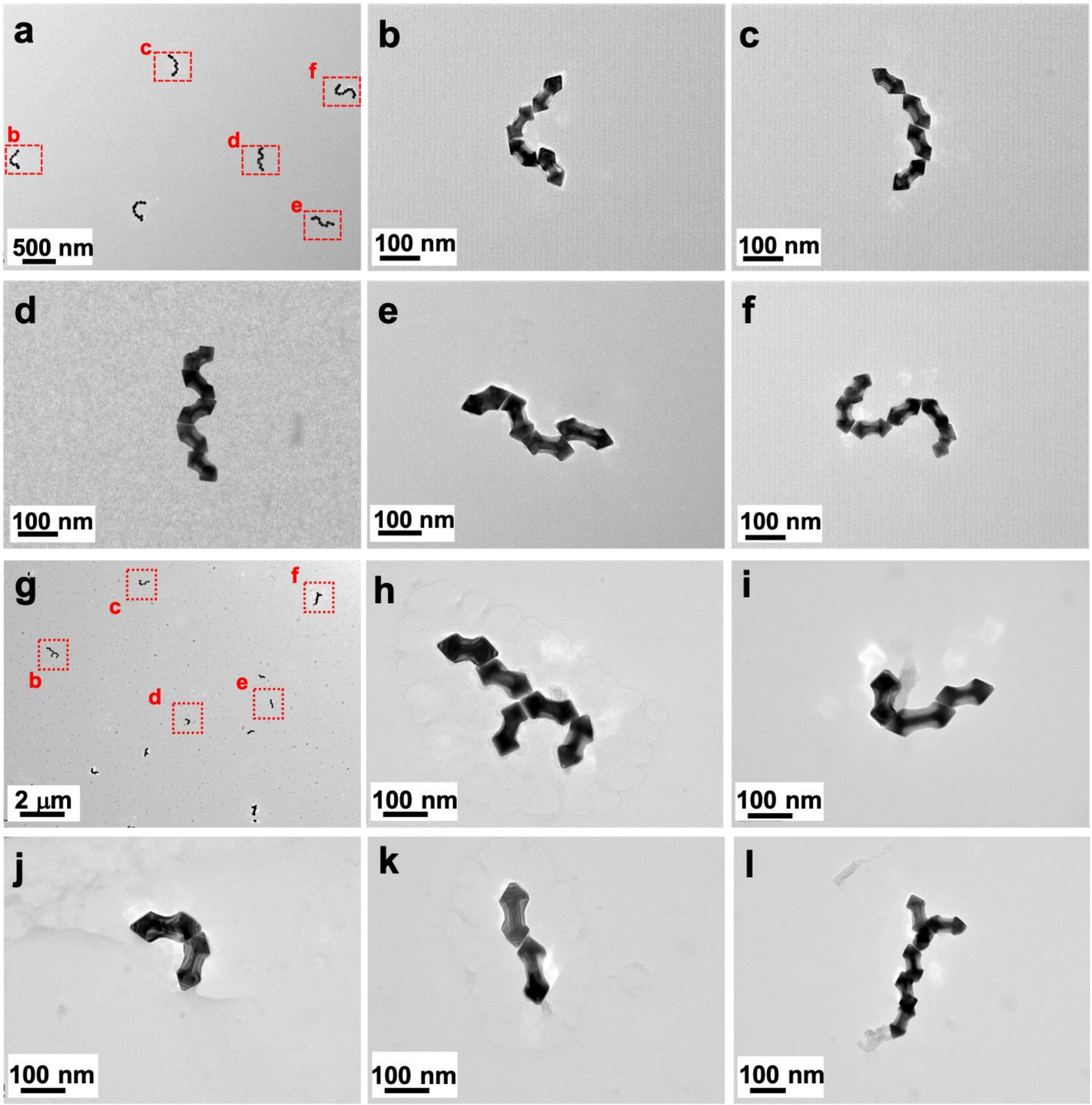

Figure S5. TEM images of GNAs-dithiol-40. Panels b-f present the high-magnification images of the framed areas in panel a, and panels h-l present the high- magnification images of the framed areas in panel g.

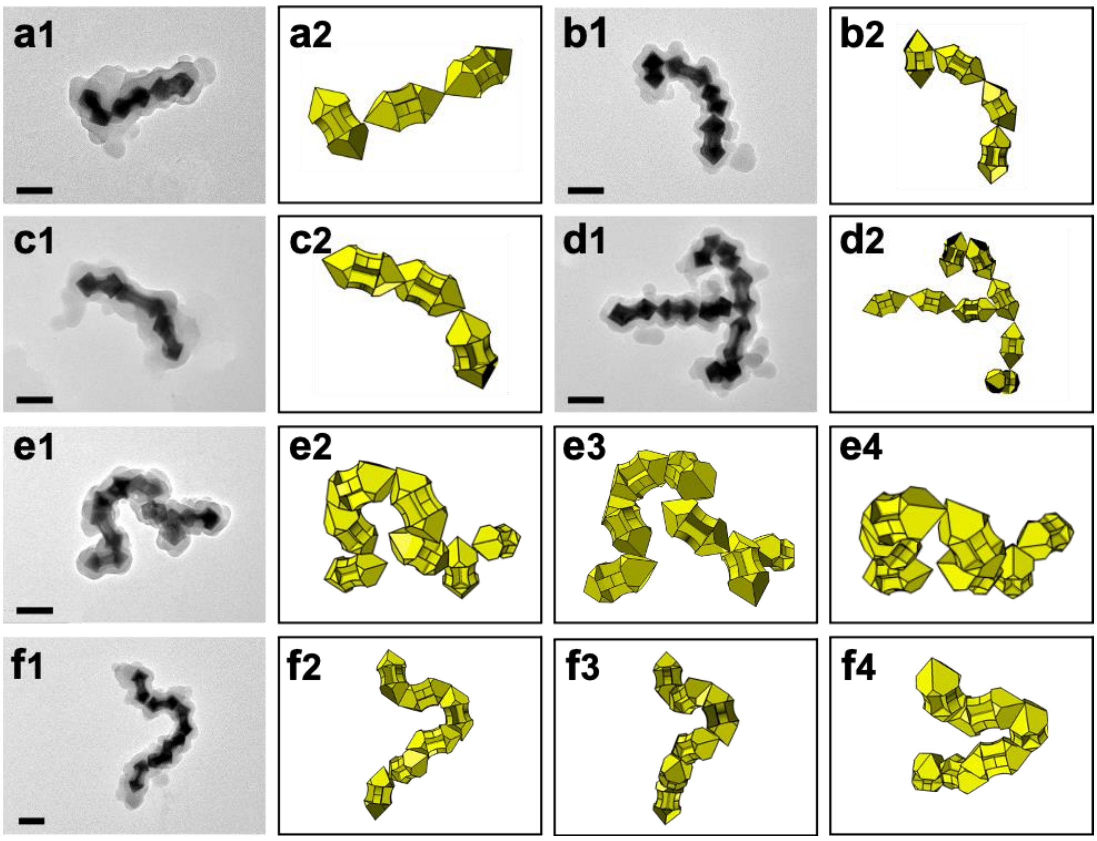

Figure S6. TEM images (a1, b1, c1, d1, e1 and f1) and geometric models (a2, b2, c2, d2, e2-4, and f2-4) of one-step dithiol-induced GNA assemblies after silica coating: (a,b) GNAs-dithiol-5, (c,d) GNAs-dithiol-20, (e,f) GNAs-dithiol-40. Scale bar: 100 nm.

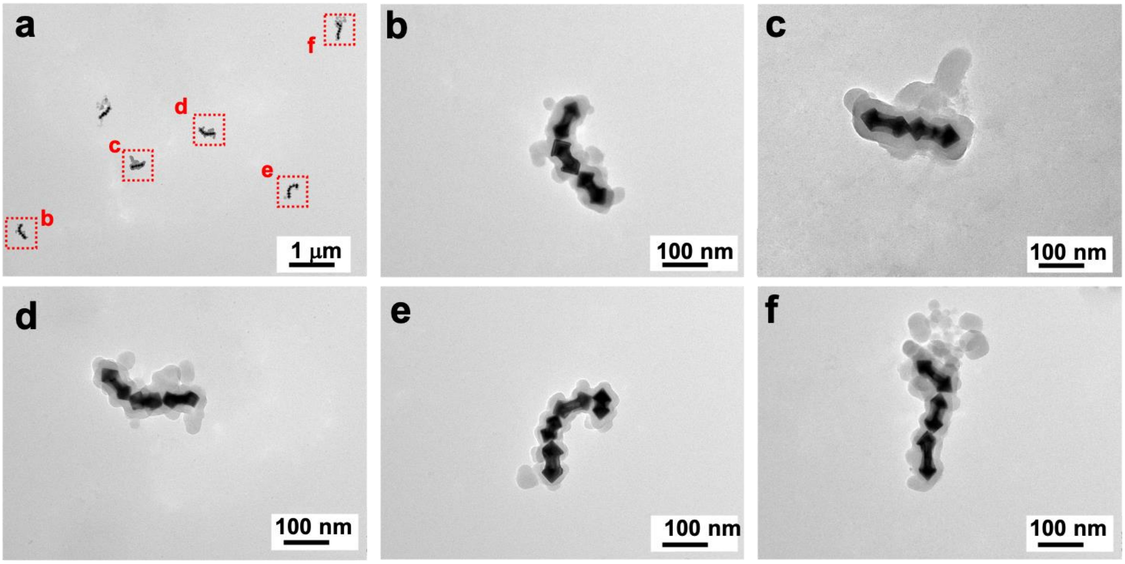

Figure S7. TEM images of silica-coated GNAs-dithiol-5. Panels b-f present the high-magnification images of the framed areas in panel a.

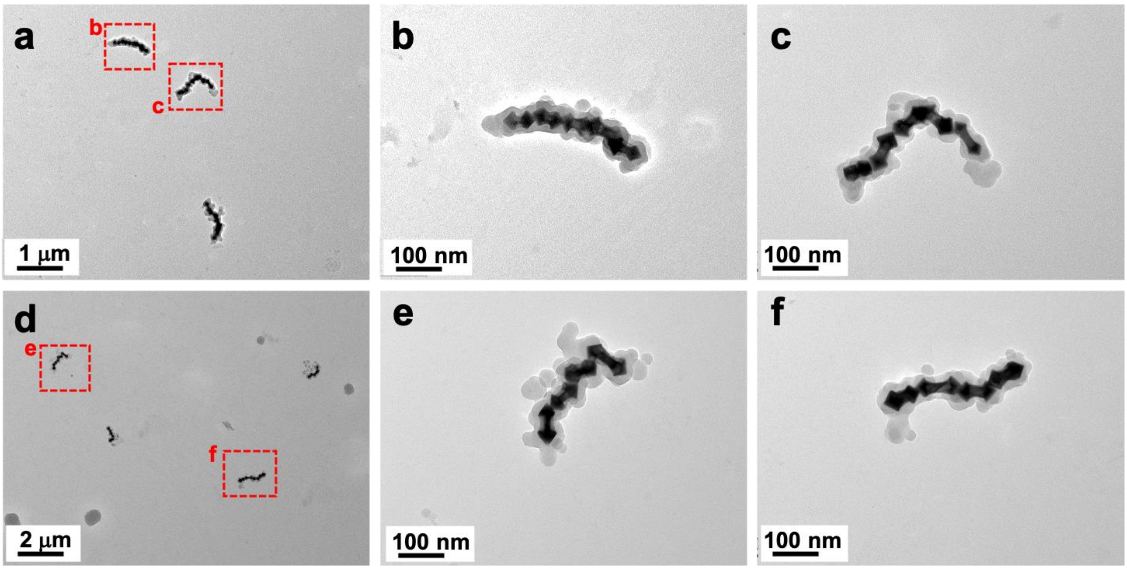

Figure S8. TEM images of silica-coated GNAs-dithiol-20. Panels b and c present the high-magnification images of the framed areas in panel a; Panels e and f present the high- magnification images of the framed areas in panel d.

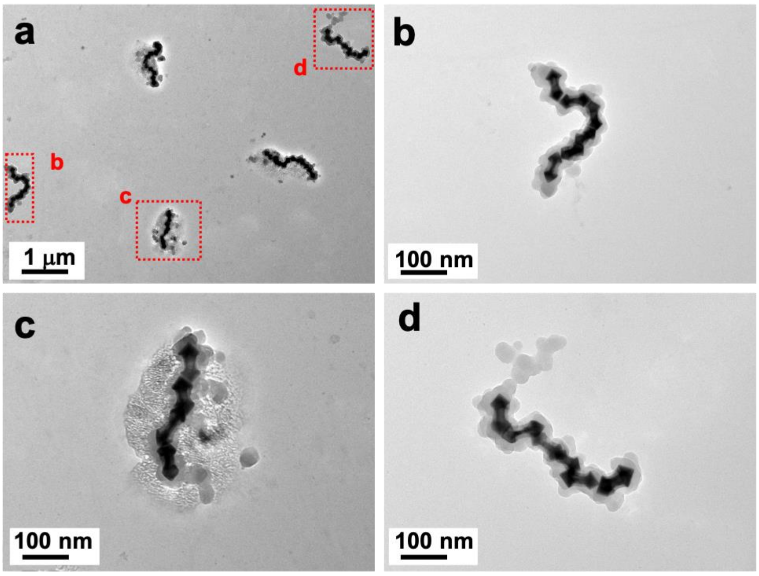

Figure S9. TEM images of silica-coated GNAs-dithiol-40. Panels b-d present the high-magnification images of the framed areas in panel a.

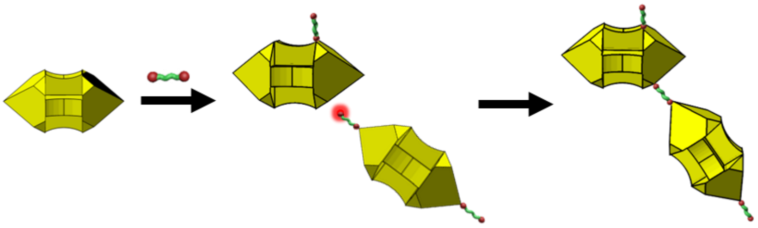

Figure S10. Schematic illustration of one-step dithiol-induced self-assembly of GNAs in a top vertex-to-side vertex mode.

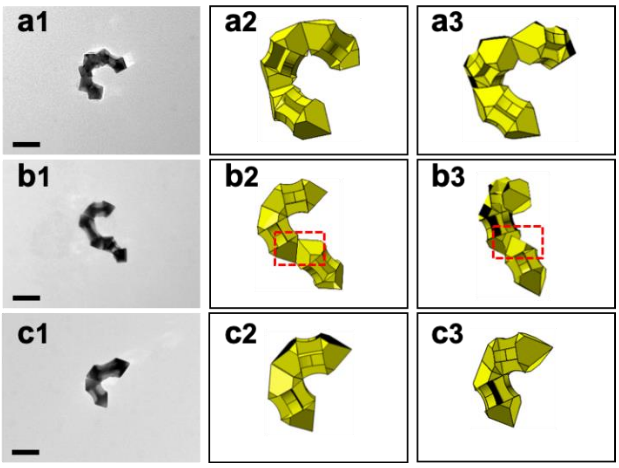

Figure S11. TEM images (a1, b1, and c1) and geometric models (a2-3, b2-3, and c2-3) of edge-to-edge (a), edge-to-facet (b), and facet-to-facet (c) assemblies obtained by two-step thiol-dithiol-induced self-assembly of GNAs. Framed boxes highlight the specific assembly mode. Scale bar: 100 nm.

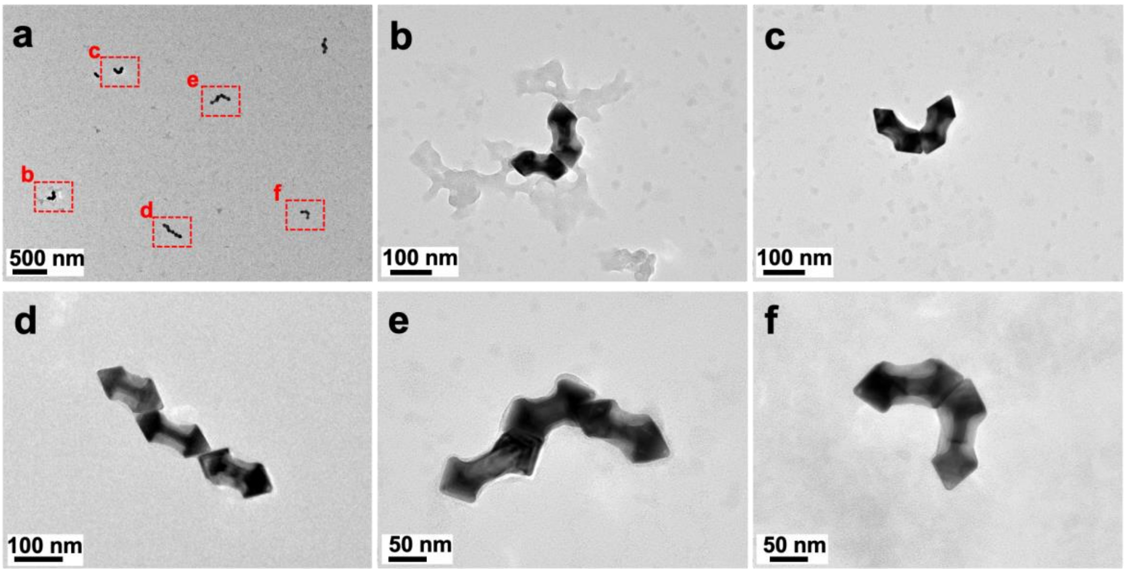

Figure S12. TEM images of GNAs-thiol-20-dithiol. Panels b-f present the high-magnification images of the framed areas in panel a.

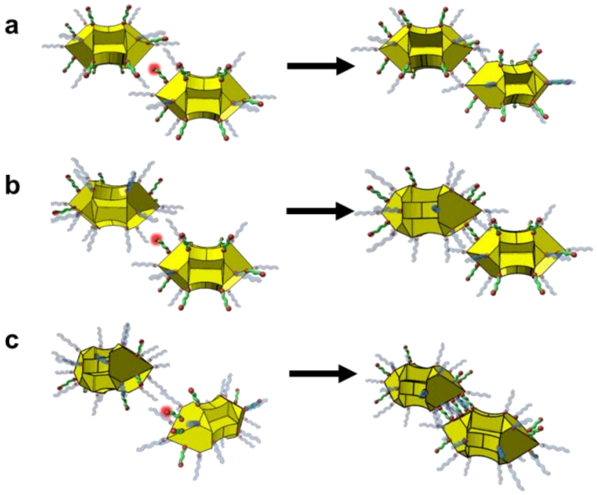

Figure S13. Schematic illustration of assembly mechanisms of two-step thiol-dithiol-induced self-assembly: (a) edge-to-edge, (b) edge-to-facet, and (c) facet-to-facet. For simplicity, the adsorbed CTAB molecules are omitted. The thiol groups to bond to another GNA are highlighted with red halos.

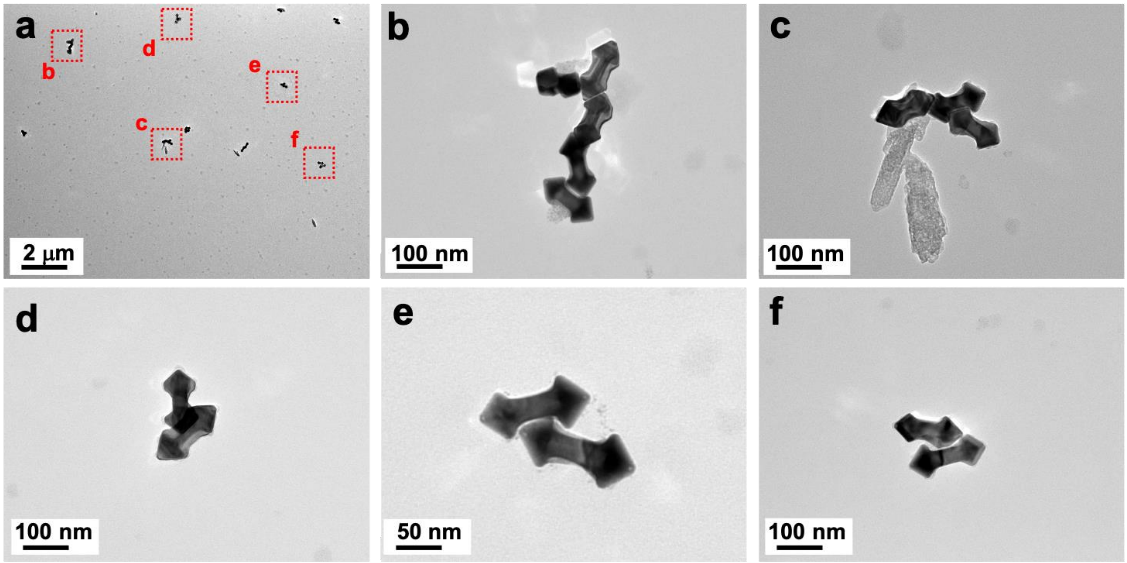

Figure S14. TEM images of GNAs-thiol-40-dithiol. Panels b-f present the high-magnification images of the framed areas in panel a.

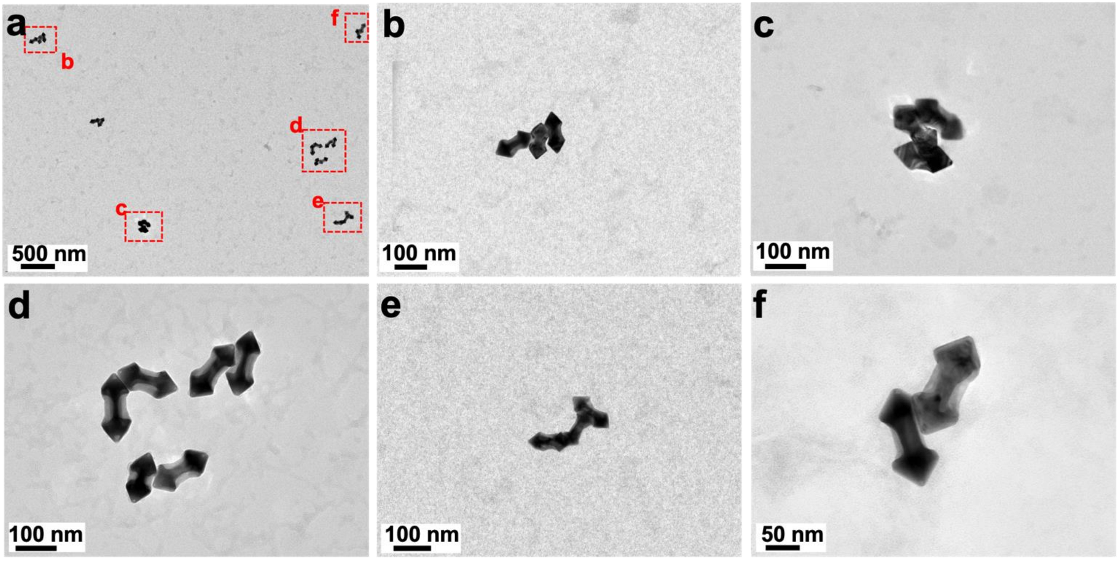

Figure S15. TEM images of GNAs-thiol-60-dithiol. Panels b-f present the high-magnification images of the framed areas in panel a.

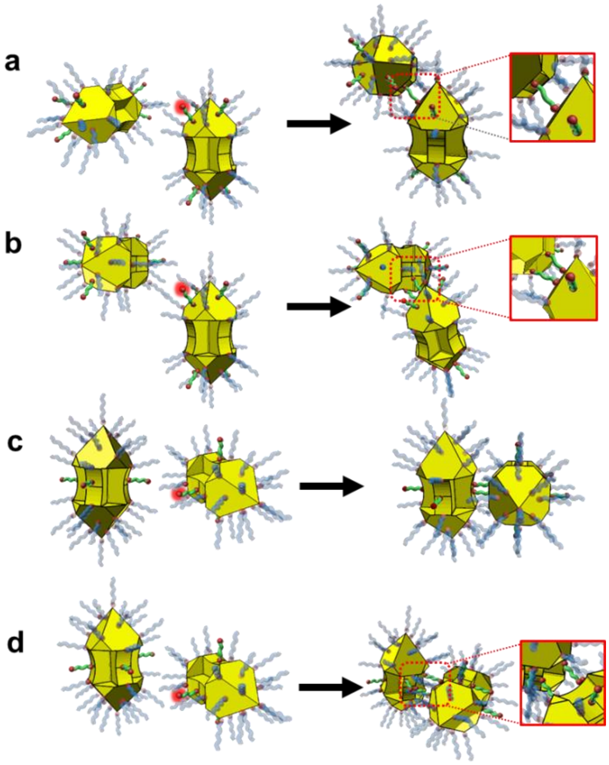

Figure S16. Schematic illustration of assembly mechanisms of two-step thiol-dithiol-induced self-assembly: (a) facet-to-one-wing, (b) facet-to-two-wings, (c) wing-to-one-wing, and (d) wing-to-two-wings. For simplicity, the adsorbed CTAB molecules are omitted. The thiol groups to bond to another GNA are highlighted with red halos.

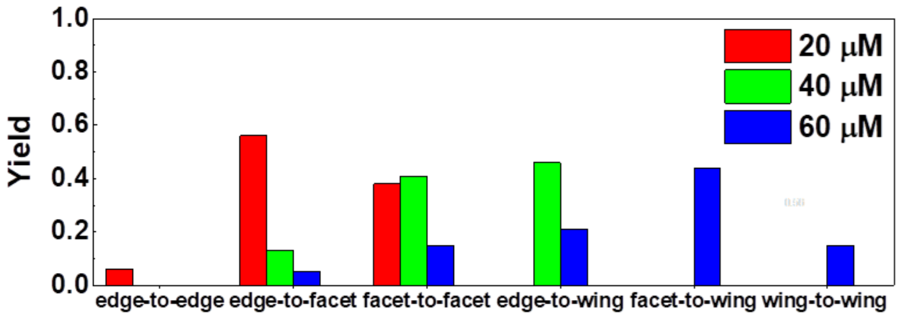

Figure S17. Statistical analysis of yield of assembly modes for two-step thiol-dithiol-induced self-assembly at different thiol concentrations.
